# Supplementary material for: Cell division machinery drives cell-specific gene activation during bacterial differentiation
Source: bioRxiv. 2023 Aug 10:2023.08.10.552768. Preprint. [Version 1] doi: 10.1101/2023.08.10.552768 (PMC10542145; doi:10.1101/2023.08.10.552768)
Supplement: 1 [file NIHPP2023.08.10.552768V1-supplement-1.pdf]

bioRxiv preprint doi: https://doi.org/10.1101/2023.08.10.552768; this version posted August 10, 2023. The copyright holder for this preprint (which was not certified by peer review) is the author/funder. This article is a US Government work. It is not subject to copyright under 17 USC 105 and is also made available for use under a CC0 license.

SUPPLEMENTAL MATERIAL

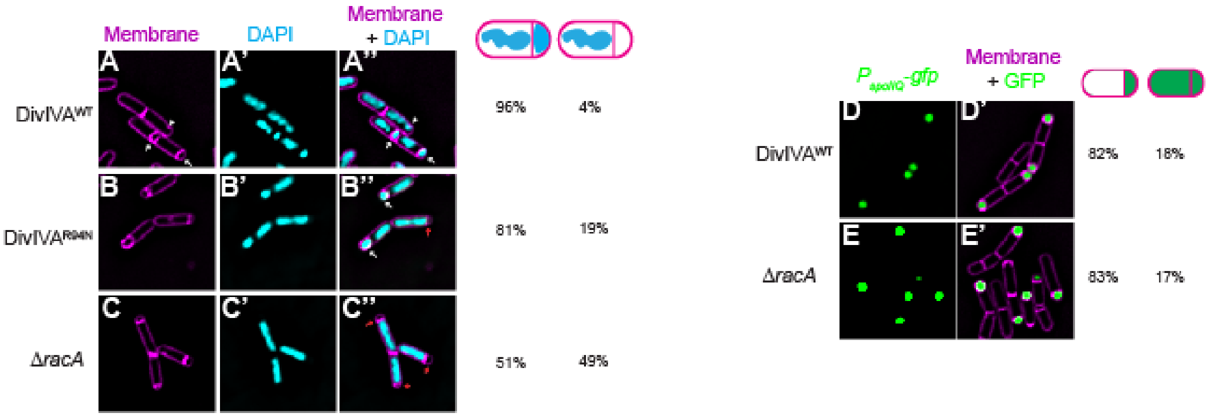

Figure S1. DivIVA^{R94N} does not cause a defect in chromosome anchoring at the onset of sporulation. (A) Fluorescence micrographs of sporulating cells of *B. subtilis* producing (A-A'') WT DivIVA, (B-B'') DivIVA^{R94N}, or (C-C'') harboring a deletion of *racA* imaged 1.5 h after the onset of sporulation. (A-C) Membranes visualized using FM4-64; (A'-C') chromosomes visualized using DAPI; (A''-C'') overlay, membranes and chromosome. (E-E') Fluorescence micrographs monitoring σ^F activation using promoter fusions (P_{spoIIQ}-*gfp*, a σ^F-controlled promoter) in (D-D') otherwise WT cells or (E-E') cells harboring deletion of *racA* at t = 1.5 h after induction of sporulation. D-E: Fluorescence from GFP production; D'-E': overlay, GFP and membranes visualized using FM4-64.

bioRxiv preprint doi: https://doi.org/10.1101/2023.08.10.552768; this version posted August 10, 2023. The copyright holder for this preprint (which was not certified by peer review) is the author/funder. This article is a US Government work. It is not subject to copyright under 17 USC 105 and is also made available for use under a CC0 license.

Table S1. *Bacillus subtilis, Escherichia coli* strains and plasmids used in this study.

Strains

Name	Genotype	Source
PY79	Prototrophic derivative of *B. subtilis* 168	(1)
KR546	Δ*divIVA::erm*	(2)
BRAF22	Δ*divIVA::erm thrC::divIVA spec*	This study
APB8	Δ*divIVA::erm thrC::divIVA*^{R94N} *spec*	This study
SJC124	Δ*divIVA::cm thrC::divIVA spec sacA::*P_{spoIIQ}*-GFP kan*	This study
SJC93	Δ*divIVA::cm thrC::divIVA*^{R94N} *spec sacA::*P_{spoIIQ}*-GFP kan*	This study
SC634	Δ*divIVA::erm* Δ*spoIID* Δ*spoIIM thrC::divIVA spec amyE::divIVA GFP cat*	This study
SC635	Δ*divIVA::erm* Δ*spoIID* Δ*spoIIM thrC::divIVA*^{R94N} *spec amyE::divIVA*^{R94N}*-GFP cat*	This study
SC656	Δ*divIVA::erm* Δ*spoIID* Δ*spoIIM* Δ*spoIIQ thrC::divIVA spec spoIIE-GFP kan*	This study
SC657	Δ*divIVA::erm* Δ*spoIID* Δ*spoIIM* Δ*spoIIQ thrC::divIVA*^{R94N} *spec spoIIE-GFP kan*	This study
SJC112	Δ*divIVA::erm thrC::divIVA*^{R94N} *spec amyE::*P_{ftsA}*-ftsAZ cat*	This study
SJC125	Δ*divIVA::erm thrC::divIVA*^{R94N} *spec sacA::*P_{spoIIQ}*-GFP kan amyE::*P_{ftsA}*-ftsAZ cat*	This study
SC527	Δ*divIVA::erm thrC::divIVA*^{R94N} *spec amyE::*P_{ftsA}*-ftsA cat*	This study
SC529	Δ*divIVA::erm thrC::divIVA*^{R94N} *spec sacA::*P_{spoIIQ}*-GFP kan amyE::*P_{ftsA}*-ftsA cat*	This study
SC544	Δ*divIVA::erm thrC::divIVA*^{R94N} *spec amyE::*P_{ftsA}*-ftsZ cat*	This study
SC546	Δ*divIVA::erm thrC::divIVA*^{R94N} *spec sacA::*P_{spoIIQ}*-GFP kan amyE::*P_{ftsA}*-ftsZ cat*	This study
SC688	Δ*divIVA::erm* Δ*spoIID* Δ*spoIIM thrC::divIVA*^{R94N} *spec amyE::divIVA*^{R94N}*-GFP cat sacA::ftsAZ cat::tet*	This study
SC671	Δ*divIVA::erm* Δ*spoIID* Δ*spoIIM thrC::divIVA*^{R94N} *spec spoIIE-GFP kan sacA::ftsAZ cat::tet*	This study
BTH101	*E.coli F' , cya-99, araD139, galE15, galK16, rpsL1 (StrR), hsdR2, mcrA1, mcrB1, relA1* Euromedex (ref. EUK001)	(3)

Plasmids

Name	Description	source
pKNT25	Derived from pSU40 Plac–MCS(HindIII–SphI–PstI–XbaI–BamHI–SmaI–KpnI–SacI–EcoRI)–T25	(3)
pUT18	Derived from pUC19. Plac–MCS(HindIII–SphI–PstI–SalI–XbaI–BamHI–SmaI–KpnI–SacI–EcoRI)–T18	(3)
pSC0209	SpoIIE-T18 derived from pUT18	This study

bioRxiv preprint doi: https://doi.org/10.1101/2023.08.10.552768; this version posted August 10, 2023. The copyright holder for this preprint (which was not certified by peer review) is the author/funder. This article is a US Government work. It is not subject to copyright under 17 USC 105 and is also made available for use under a CC0 license.

pSC0207	DivIVA^{WT}-T18 derived from pUT18	This study
pSC0210	DivIVA^{WT}-T25 derived from pKNT25	This study
pSC0211	DivIVA^{R94N}-T25 derived from pKNT25	This study

1. Youngman P, Perkins JB, Losick R. Construction of a cloning site near one end of Tn917 into which foreign DNA may be inserted without affecting transposition in *Bacillus subtilis* or expression of the transposon-borne erm gene. *Plasmid*. 1984;12: 1-9.
2. Eswaramoorthy P., Erb LM, Gergory AJ, Silverman J, Pogliano K, Pogliano J and Ramamurthi KS, Cellular architecture mediates DiviVA ultrastructure and regulates min activity in *bacillus subtilis. mBio* 2011
3. Battesti and Bouveret, the bacterial two-hybrid system based on adenylate cyclase reconstitution in Escherichia coli, Methods, 2012
